# Supplementary material for: Accuracy of triggering receptor expressed on myeloid cells 1 in diagnosis and prognosis of acute myocardial infarction: a prospective cohort study
Source: PeerJ. 2021 Jun 22;9:e11655. doi: 10.7717/peerj.11655 (PMC8231339; doi:10.7717/peerj.11655)
Supplement: Supplemental Information 1 — *P < 0.05,**P < 0.01. [file peerj-09-11655-s001.docx]

Supplemental table 1 Expression of TLR4 and TREM1 in normal and AMI groups. *P<0.05，**P<0.01.

| Biomarkers | Normal group (n=16) | AMI group (n=60) | P value |
| --- | --- | --- | --- |
| TLR4(ng/ml) | 3.7±1.4 | 3.6±1.7 | 0.726 |
| TREM1(pg/ml) | 127.1±50.1 | 180.2±70.0 | 0.002^**^ |
